# Combined with the first dorsal (plantar) metatarsal artery pedicle free bilobed flap with a cell scaffold for the repair of a mid-distal adjacent finger defect: a retrospective study

**DOI:** 10.1186/s13018-024-04656-5

**Published:** 2024-04-27

**Authors:** Meng Ge, Zhijin Zhang, Guohua Ren, Shenghu Hong, Cheng chen, Jun Yang, Qiao Hou, Hongmei Fu

**Affiliations:** 1 Research Institute of Orthopedics, Jiangnan Hospital Affiliated Zhejiang Chinese Medical University, Hangzhou, 312001 China; 2 Hangzhou Xiaoshan Hospital of Traditional Chinese Medicine, Hangzhou, 312001 China

**Keywords:** Free bilobed flap, First dorsal (plantar) metatarsal artery pedicle, Cell scaffold, a mid-distal adjacent finger defect, Microsurgery

## Abstract

**Purpose:**

Assessing the clinical effectiveness of combining with the first dorsal (plantar) metatarsal artery pedicle free bilobed flap with a cell scaffold to repair mid-distal defects in adjacent fingers.

**Methods:**

From September 2012 to April 2022, 21 patients with 42 mid-distal defects of adjacent fingers underwent treatment using combined with the first dorsal (plantar) metatarsal artery pedicle free bilobed flap with a cell scaffold. The flaps size ranged from 2.1 cm * 1.6 to 4.9 cm * 3.2 cm. Follow-up evaluations included assessing function, sensation, and appearance, etc. of the injured fingers and donor areas.

**Results:**

All 42 flaps survived in 21 patients without any vascular crises, and the wounds healed in phase I. The mean follow-up time was 12.2 months (range 7–22 months). During follow-up, in injured fingers, according to the Michigan Hand Outcomes Questionnaire (MHOQ), the functional recovery and appearance were satisfactory; in Dargan Function Evaluation (DFE), the results were both “excellent” in fourteen patients, “excellent” and “good” in five patients, both “good” in one patient, “good” and “general” in one. In static two-point discrimination (2PD), the variation ranges from 4 to 9 mm in injured fingers and 6—10 mm in donor toes. Cold Intolerance Severity Score (CISS) is mild in all patients. The visual analogue score (VAS) showed no pain in the injured fingers and donor toes. No deformities or other complications were noted at the donor toes. According to Chinese Manchester Foot Pain and Disability Index (C-MFPDI), there was no morbidity on foot function in all donor areas.

**Conclusion:**

The surgical procedure of combined with the first dorsal (plantar) metatarsal artery pedicle free bilobed flap with a cell scaffold for the repair of mid-distal adjacent fingers defect is highly satisfactory. This approach helps the injured fingers to achieve good function, sensibility and appearance, while also achieving satisfactory results in the donor toes.

## Introduction

In recent years, China’s industrial and technological level has rapidly developed, leading to increased incidence of hand trauma despite the facilitation of people’s daily working lives. The number of people with defects in the mid-distal area of adjacent fingers is also high [1–3]. The unique skin texture of the fingers often leading to exposure of tendons and bones, causing poor appearance and severe loss of sensation and function. Skin grafting alone does not provide adequate blood supply, necessitating the use of flaps for treatment [4–6]. However the treatment of mid-distal finger defects has long posed a challenge for hand surgeons. With the in-depth study of clinical anatomy and the development of microsurgical techniques, various methods for repairing soft tissue defects of the hand have emerged. These methods include adjacent flaps such as V-Y flaps, adjacent finger flaps, and island flaps of the intrinsic arteries on the metacarpal side of the finger, as well as distal ones like abdominal flaps, free limb miniature flaps, and free flaps of the foot. The best treatment options remain controversial due to the varying injury types and extents [7–13].

Due to the homologous nature of toe and finger skin tissues, finger defects are often repaired by tissue flap transplantation from the foot. The use of free flaps from the fibular side of the toe or the tibial side of the 2nd toe to repair finger defects can maximize the restoration of finger shape and fine sensation, while minimizing damage to the donor foot [14–16]. However, for mid-distal defects of adjacent fingers, it is often necessary to combine multiple methods or repeat the use of a method, thereby increasing the time and difficulty of surgery. Scholars have attempted to address this by designing the free first dorsal metatarsal artery pedicle bilobed flap, which has shown promising results. Although the method had become a classic technique for repairing the proximal skin and soft tissue defects of two adjacent fingers, it presents significant challenges in repairing the mid-distal defects, certain problems with the handling of the skin in the donor toes either. Many scholars have attempted to improve this technique, but with unsatisfactory results. Therefore, there is an urgent need for a surgery that is less invasive, shorter, and less difficult, and that takes into account the function, sensibility, and appearance of both the injured finger and the donor toes [17, 18].

Based on clinical and previous experience, satisfactory results were obtained in the application of cell scaffolds for the treatment of hand and foot injuries, and the use of skin grafting the pedicle of the island flap wounds in the distal area breaks the conventional practice of requiring the vascular pedicle flap to be buried under the skin [19–21]. Inspired by both the approaches, we designed combined with the first dorsal (plantar) metatarsal artery pedicle free bilobed flap with a cell scaffold for the repair of a mid-distal adjacent finger defect, good results were obtained for both the injured fingers and the donor toes. So we are currently conducting a retrospective study to use more scientific approaches to examine the clinical effectiveness of this operation, aiming at provide hand surgeons with a novel treatment modality for repairing mid-distal finger defects of adjacent fingers.

## Materials and methods

### General information

Between September 2012 and April 2022, 21 patients (thirteen males and eight females) with mid-distal defects in two adjacent fingers were treated at our hospital. The treatment involved combined with the first dorsal (plantar) metatarsal artery pedicle free bilobed flap with a cell scaffold for the repair of a mid-distal adjacent finger defect. The patients, with an average age of 45.4 years (ranging from 25 to 58), had defects in various fingers: twelve cases in the middle and ring fingers, six in the middle and index fingers, and three in the ring and little fingers. The cases included eleven instances of postoperative soft tissue necrosis due to machine crush injuries, six cases due to thermal compression injuries, and four cases of cuts. After debridement, the finger defects ranged from 1.9 cm × 1.4 to 4.7 cm × 3.0 cm, while the free flaps ranged from 2.1 cm × 1.6 to 4.9 cm × 3.2 cm. Average Surgery time is 197 min ranged from 180 to 230 min (Table 1). All patients underwent preoperative workup, including anteroposterior and oblique X-rays to rule out finger fractures.

**Table 1:** Patient characteristics and operation time

Case	Age	Sex	Injured finger	Injury type	Defect size(cm)	Flap size(cm)	Operation time(min)	Follow-up(month)
1	25	M	RMARF	thermal compression injuries	1.7 × 1.6/ 2.0 × 1.6	1.9 × 1.8/ 2.2 × 1.8	185	8
2	53	M	LMARF	machine crush injuries	3.6 × 1.5/3.0 × 1.8	3.8 × 1.7/3.2 × 2.0	210	7
3	52	F	RMARF	cut	2.2 × 1.6/1.9 × 1.8	2.4 × 1.8/2.1 × 2.0	195	10
4	54	F	LMARF	thermal compression injuries	1.9 × 1.4/ 2.8 × 1.8	2.1 × 1.6/ 3.0 × 2.0	190	12
5	51	F	LMARF	machine crush injuries	4.8 × 2.3/ 4.6 × 2.6	5.0 × 2.5/ 4.8 × 2.8	215	14
6	41	M	RMAIF	machine crush injuries	3.4 × 2.6/ 2.7 × 2.3	3.8 × 2.8/ 3.5 × 2.5	190	7
7	52	M	LMARF	thermal compression injuries	1.8 × 1.5/1.9 × 1.6	2.0 × 1.7/2.1 × 1.8	180	10
8	45	F	LMARF	machine crush injuries	3.5 × 2.0/ 3.2 × 1.8	3.7 × 2.2/ 3.6 × 2.0	200	20
9	56	F	RMAIF	machine crush injuries	4.7 × 3.0/ 4.2 × 2.8	4.9 × 3.2/ 4.4 × 3.0	200	9
10	37	M	LMARF	cut	2.4 × 1.4/1.8 × 1.8	2.6 × 1.6/2.0 × 2.0	215	11
11	43	M	RMARF	cut	3.0 × 1.7/4.2 × 2.4	3.2 × 1.9/4.4 × 2.6	210	22
12	36	M	LRALF	machine crush injuries	4.4 × 2.6/4.2 × 1.5	4.6 × 2.8/4.4 × 1.7	185	8
13	38	M	LMAIF	machine crush injuries	4.4 × 1.4/ 4.0 × 1.5	4.6 × 1.6/4.2 × 1.7	230	18
14	32	F	RMARF	machine crush injuries	2.2 × 1.6/2.0 × 1.6	2.4 × 1.8/2.2 × 1.8	190	12
15	48	F	LMAIF	thermal compression injuries	1.9 × 1.9/2.0 × 1.8	2.1 × 2.1/2.2 × 2.0	180	10
16	49	M	LMARF	machine crush injuries	4.0 × 2.1/ 3.8 × 1.8	4.2 × 2.3/4.0 × 2.0	190	9
17	55	M	LMARF	machine crush injuries	3.4 × 1.9/ 3.2 × 1.8	3.6 × 2.1/3.4 × 2.0	210	9
18	53	F	RMARF	machine crush injuries	3.0 × 2.0/ 4.0 × 1.5	3.2 × 2.2/4.2 × 1.7	200	17
19	40	F	RMARF	cut	3.0 × 1.7/4.2 × 2.4	3.2 × 1.9/4.4 × 2.6	190	14
20	35	M	LMAIF	thermal compression injuries	2.6 × 1.8/ 2.8 × 1.9	2.8 × 2.0/ 3.0 × 2.1	180	19
21	58	M	LMAIF	machine crush injuries	3.8 × 1.7/3.2 × 2.0	4.0 × 1.9/3.4 × 2.2	190	10

LMADF: Left middle and index fnger RMADF:Right middle and index finger

RMARF: Right middle and ring finger LMARF : Left middle and ring finger

LRALF: Left ring and little finger; M: Male; F: Female

### Cell scaffold

**Fig. 1:**
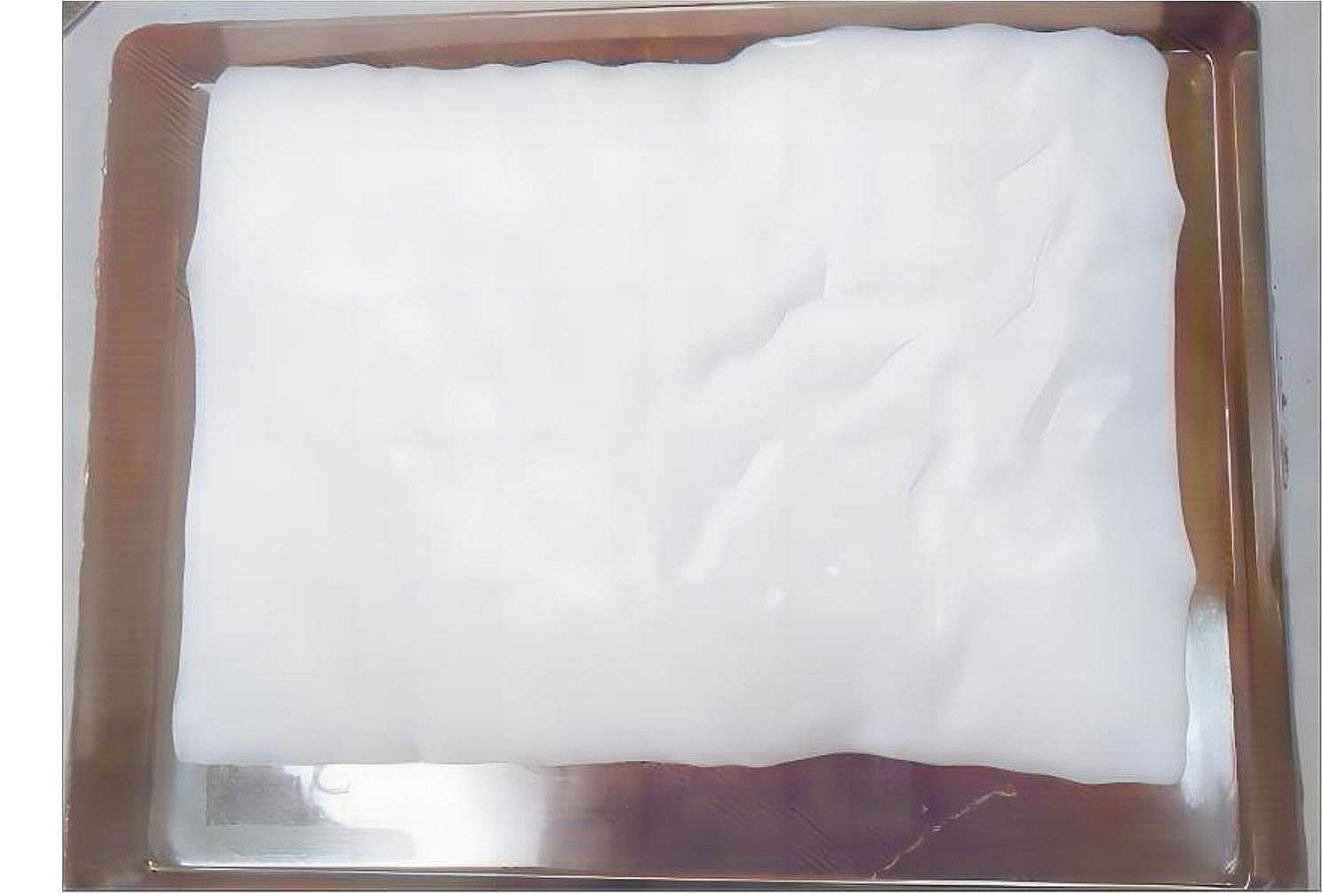
Physical diagram of the cell scaffold

The cell scaffold (Pelnac) we applied is produced by GUNZE limited. The cell scaffold is designed to temporarily replace dermal tissue and promote healing. Its unique structure, with a silicone outer layer and collagen inner layer, allows for high histocompatibility and low antigenicity. The use of pig tendon-derived collagen processed into terminal-free collagen ensures a biocompatible material for medical applications. This scaffold provides a supportive framework for cells to grow and regenerate, making it a valuable tool in tissue engineering and wound healing [21, 22]. (Fig. 1).

#### Inclusion criteria

(1) Patients aged between 15 and 60, sober, without psychological disorders, and willing to participate in treatment and follow-up. (2) Skin and soft tissue defects in the mid-distal segments of two adjacent fingers with an area of more than 1 square centimeter. (3) Patients with a strong desire to preserve the finger and restore sensation, function, and appearance. (4) Patients with no history of previous injury to the affected finger.

#### Exclusion criteria

(1) Age below 15 or above 60. (2) Single finger injury or injury to more than 2 fingers. (3) Proximal injury or mid-distal defects of two adjacent fingers with an area of less than 1 square centimeter. (4) Smokers and patients with poor compliance with treatment and bed rest requirements.

This clinical study was approved by our institutional review board.

### Surgical treatment procedure

**Fig. 2:**
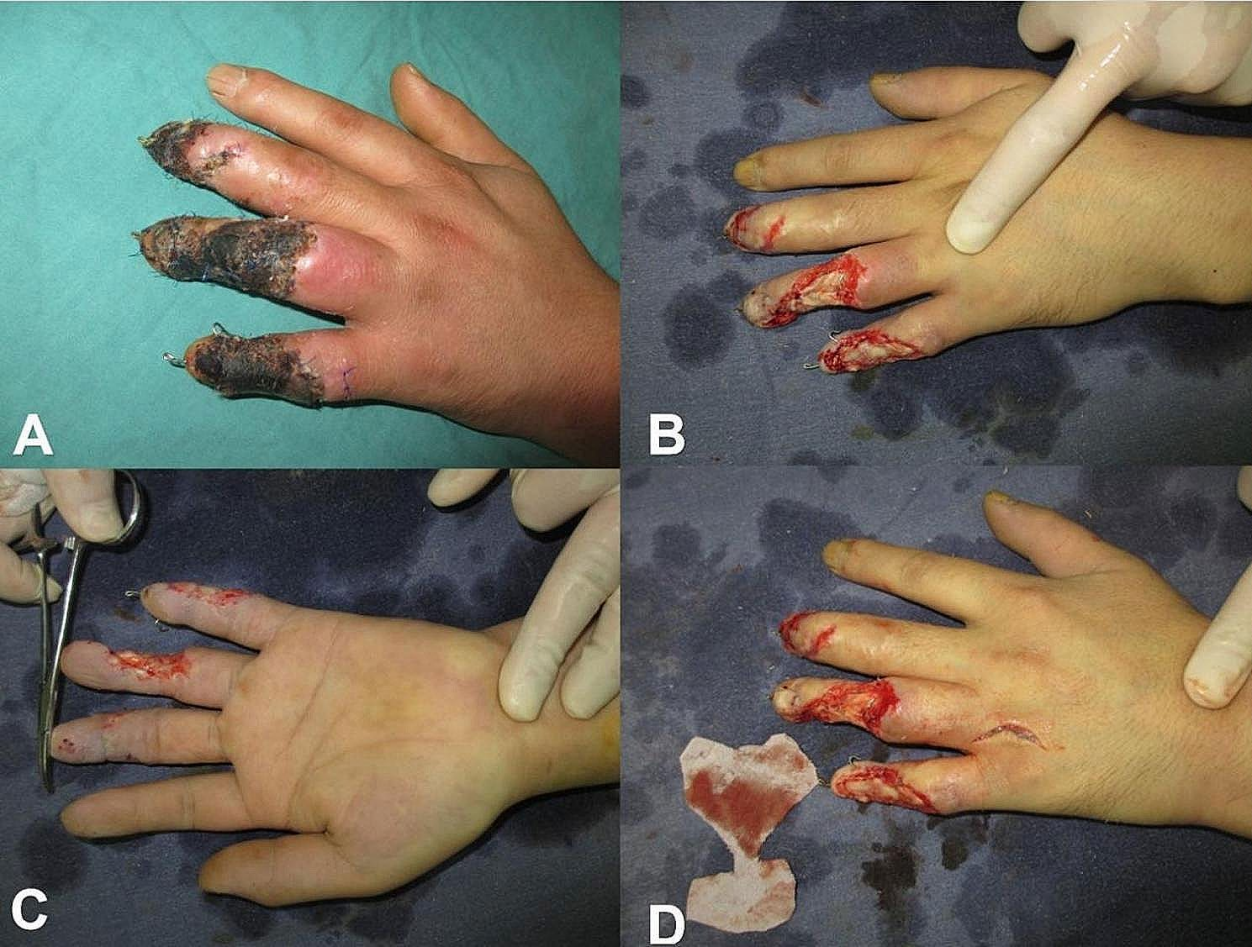
Thoroughly debride and obtain a cloth pattern of the defect shape. **A**: Skin necrosis after machine crush injury. **B-C**: Appearance after debridement surgery. **D**: Cutting cloth pattern according to the shape of the defect

Treatment of hand recipient site: The surgery was performed under brachial plexus block anesthesia combined with continuous epidural anesthesia. Balloon tourniquet were applied to the upper and lower limbs (with a pressure of 30 kPa for the upper limb and 50 kPa for the lower limb). The injured finger was debrided, removing necrotic skin and subcutaneous tissue until a rich vascular bed was formed. At one of the injured finger sites, a zigzag incision was made on the palmar side near common finger artery to open the subcutaneous tunnel and expose the normal segment of the finger artery or the common finger artery. Then, a longitudinal arc-shaped incision approximately 1–2 cm long was made between the dorsal aspects of the two injured fingers, exposing the cephalic or one dorsal vein of the hand, and a wide subcutaneous tunnel was created from the incision to the wound surface using vascular forceps, allowing it to communicate with the finger wound through the subcutaneous tunnel. Based on the external shape of the two wounds, fabric was cut and used for the design of flaps. After the fabric cutting was completed, the tourniquet was released, and the wound was thoroughly hemostatic, followed by dressing the wound with sterile gauze and bandages. (Fig. 2).

#### Flap design and cutting

The choice of the donor area of the foot: as far as possible, the defect of the larger trauma will be designed on the big toe is preferred. Based on the required size and shape of the nail bed, select a suitable donor site on the foot for harvesting. Outline the size of the skin flap according to the pattern and draw the incision line (on the same side or opposite side is optional). Once the supply area has been selected, along the flap design line of the proximal dorsal incision, and along the 1st dorsal metatarsal artery in the first, second metatarsal dorsal to make a longitudinal “S” shaped incision to the subcutaneous, then the dorsal metatarsal vein and its associated plantar dorsal vein or plantar vein arch were then routinely isolated from distal to proximal according to the flap design line. Move deeper and further apart, find the **first dorsal metatarsal artery** and its connected plantar artery of the fibular side of the toe and tibial side of the second toe When the first dorsal metatarsal artery is thin or absent, you can first find plantar artery of the fibular side of the toe and tibial side of the second toe, and make a longitudinal incision on the sole of the foot and locate the first plantar metatarsal artery proximally.

**Fig. 3:**
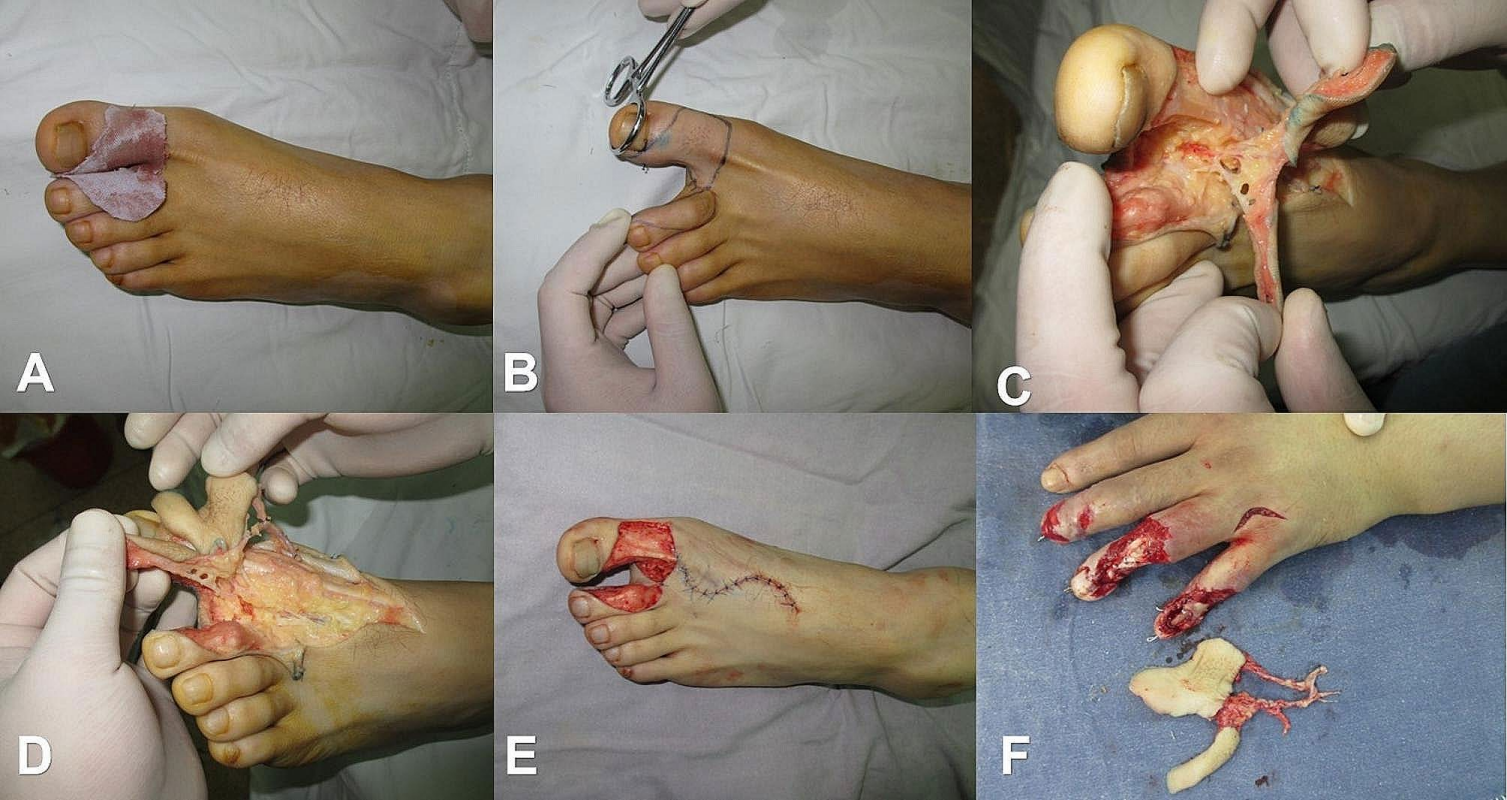
Design and harvesting of the free bilobed flap. **A-B**: Design of the free bilobed flap. **C-F**: Harvesting of the free bilobed flap

The plantar nerve of the fibular side of the great toe and the tibial side of the second toe, which run concomitantly with the plantar artery of the toe, are cut as needed. The distal and metatarsal sides of the flap are incised after the flap has been dissected along the pedicle to an appropriate length, then separate the flaps anterograde distally along nerve pedicle of the plantar internal artery respectively, with care taken to preserve part of the soft tissue in the donor area and the peritendinous membrane as much as possible. The toenail is removed using sharp dissection method for complete excision. After the flap is obtained, the tourniquet is loosened, and after the blood supply is restored, the flap pedicle is cut off to free the flap (Fig. 3).

#### Treatment of the foot donor area

After achieving complete hemostasis in the wound, the tourniquet is carefully loosened. The cell scaffold is then immersed in a 0.9% sodium chloride solution to ensure full saturation. Following this, the scaffold is meticulously trimmed to an appropriate size based on the shape of the donor area. The collagen sponge surface is delicately applied to the wound surface using separate sutures, and then slightly compressed and carefully bandaged. In cases where the defect area is large, the pressure can be increased appropriately to ensure optimal application and coverage.

#### Flap transfer process

After transferring the flap to cover the wound on the hand, secure it with 5 − 0/4 − 0 prolene sutures. Then, we anastomosed the plantar nerve with the finger nerve stumps, leading the dorsal (plantar) metatarsal artery to the palm of the hand to anastomose with the common artery of the finger and anastomose the dorsal metatarsal vein/ plantar dorsal vein or plantar vein arch to the cephalic vein or (and) dorsal vein of the hand through subcutaneous tunnels. The pedicle between the flaps is covered with the cell scaffold.

### Postoperative treatment

**Fig. 4:**
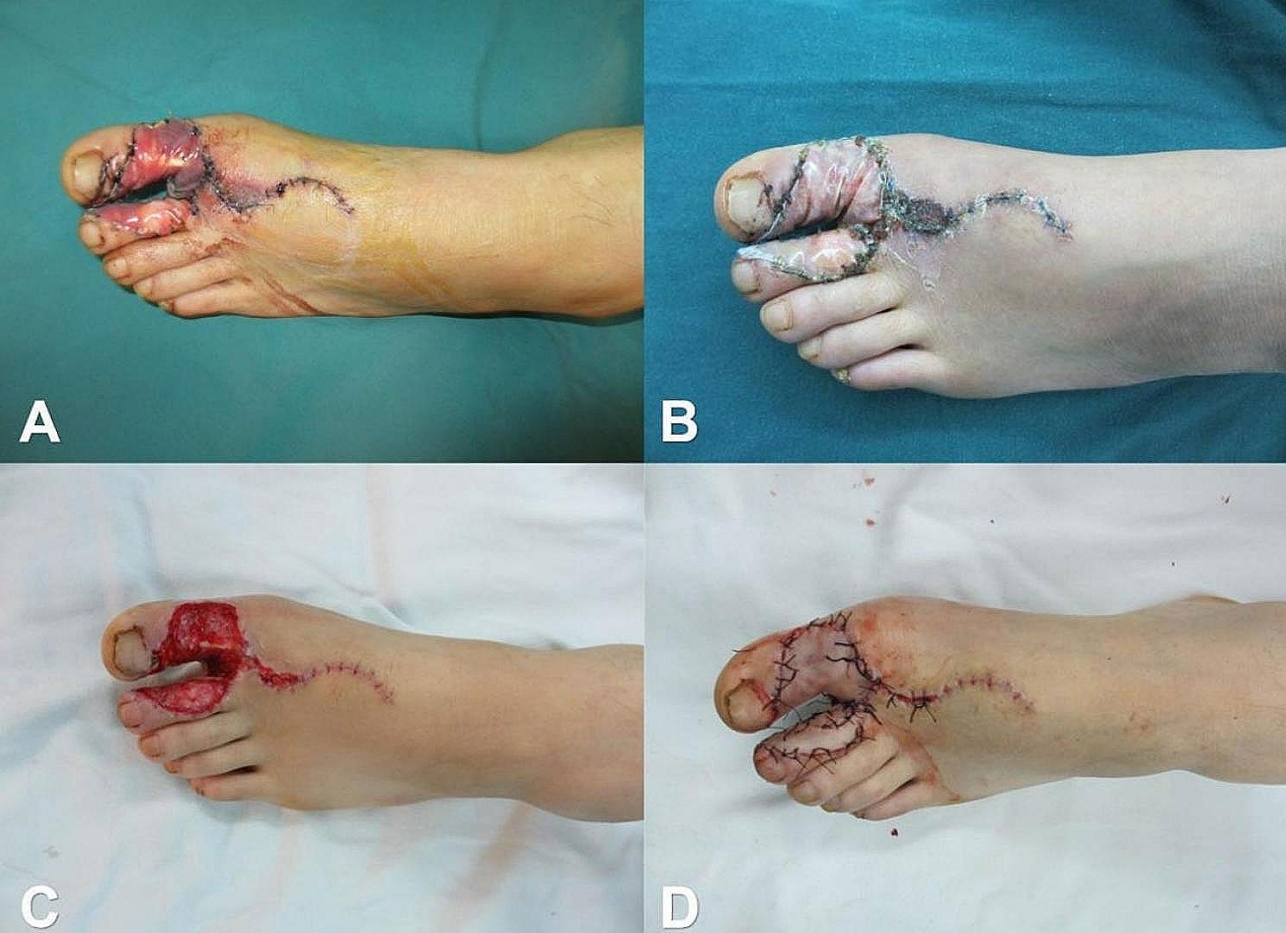
Treatment of the foot donor area. **A**: Appearance one week after cell scaffold implantation surgery. **B**: Appearance three weeks after cell scaffold implantation surgery. **C**: Appearance after removal of the cell scaffold membrane. **D**: Appearance after skin grafting

**Fig. 5:**
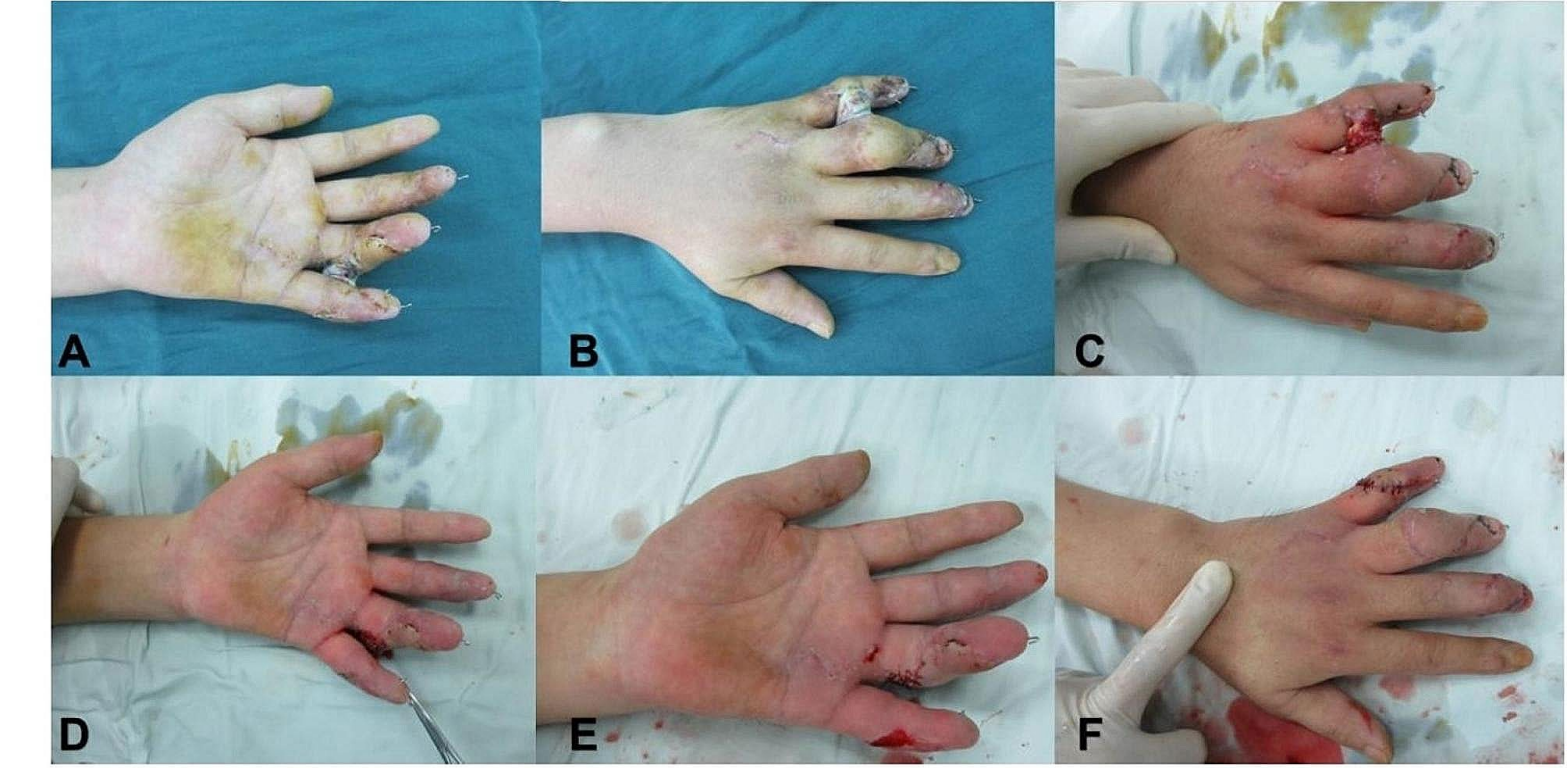
Treatment of the hand recipient area. **A-B**: Appearance one week after free bilobed flap combined with cell scaffold repair surgery. **C-D**: Appearance three weeks after surgery. **E-F**: Appearance after pedicle division surgery

After the phase I surgery, the patient received anti-inflammatory, anticoagulation, antispasmodic, and other symptomatic treatments. After 7 days post-surgery, the patient was instructed to perform hand flexion and extension functional exercises. Three weeks after the surgery, the collagen will be degraded and replaced by the new granulation tissue, forming the pink dermoid tissue seen in clinical practice, and establishing a blood circulation connection with the bone or tendon covered by it, effectively covering the wound. When the cell scaffold silicone film layer was separated from collagen sponge layer and the cell scaffold provides an adequate substrate for skin grafting, this is followed by skin graft, and then the pedicle was severed between the free flaps of the two fingers (Figs. 4 and 5).

### Observation index and evaluation method

All assessments were conducted by a senior hand surgeon not involved in the surgery. Patients were followed up for more than 6 months. During the final follow-up, we used the Michigan Hand Outcome Questionnaire (MHOQ) to assess the function and appearance of the injured fingers [23]. In this questionnaire, higher scores indicate better appearance and function. The function of the interphalangeal joints of the fingers was evaluated using the Dargan function evaluation (DFE) as follows: Excellent: flexed fingertip passes through the transverse pattern of the palm; Good: flexed fingertip reaches the transverse pattern of the palm; General: the distance between the flexed fingertip and the transverse pattern of the palm is less than 2 cm; Poor: distance between fingertip flexion and the transverse pattern of the palm is > 2 cm [24]. We used the static two-point discrimination (2PD) test to assess the sensitivity of the injured finger and donor area [25]. The home-made Cold Intolerance Severity Score (CISS) questionnaire was used to assess the degree of cold intolerance, categorized into four grades: 0–25 (mild), 26–50 (moderate), 51–75 (severe), or 76–100 (very severe) [26]. Pain levels were evaluated using a pain visual analog score (VAS) for both the injured fingers and the donor toes. The functions of the donor foot were assessed according to the Chinese Manchester Foot Pain and Disability Index (C-MFPDI), which consists of 17 questions, each divided into three levels of severity [27]. A score of 1, 2, or 3 was assigned to indicate the degree from severe to mild, with a minimum score of 17 out of 51, and higher scores representing better foot functioning.

## Results

**Fig. 6:**
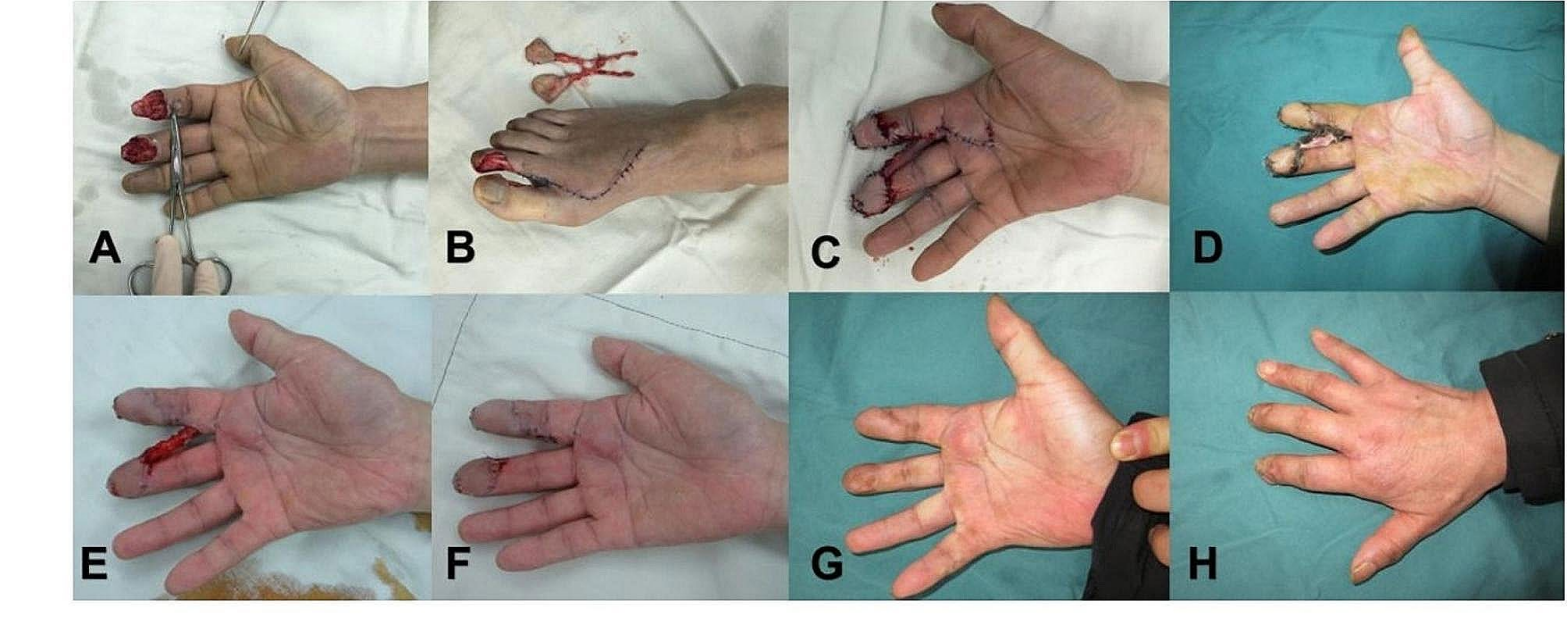
Case 6. **A**: Skin and soft tissue defects after machine crush injury. **B**: Harvested free bilobed flap. **C-D**: Appearance after free bilobed flap combined with cell scaffold repair surgery. **E**: Appearance after removal of the outer layer of the cell scaffold. **F**: Appearance after pedicle division surgery. **G-H**: Appearance six months after pedicle division surgery

**Fig. 7:**
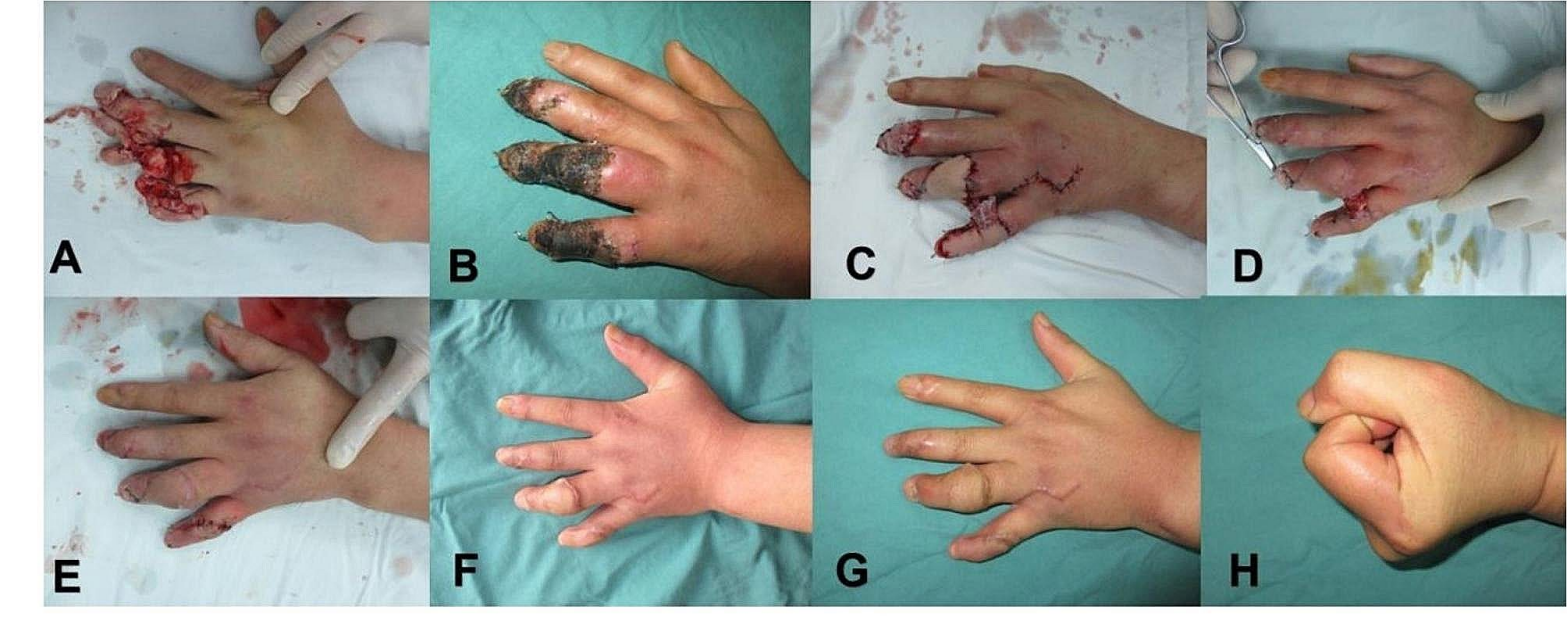
Case 12. **A**: Skin and soft tissue defects after machine crush injury. **B**: Skin necrosis. **C**: Appearance after free bilobed flap combined with cell scaffold repair surgery. **D**: Appearance after removal of the cell scaffold membrane. **E**: Appearance after pedicle division surgery. **F**: Appearance and function three months after pedicle division surgery. **G-H**: Appearance and function eight months after pedicle division surgery

All 42 flaps were successful in 21 patients, with no vascular crises occurring in any of the bilobed flap cases, and the wounds healing in one stage. The average patient follow-up was 12.2 months (range 7–22 months). During follow-up, the functional recovery and appearance of the reconstructed fingers, as assessed by the MHOQ, were found to be satisfactory. In the DFE, both “excellent” in fourteen patients, “excellent” and “good” in five patients, both “good” in one patient, “good” and “general” in one. The static 2PD ranged from 4 to 9 mm, and the CISS was mild in all patients, with the VAS showing no pain in the reconstructed finger. At the final follow-up, no deformities or complications were observed at the donor site, and the static 2PD ranged from 6 to 10 mm. According to the VAS results, there was no residual pain in the donor foot. All patients scored 51 points in the C-MFPDI, indicating no adverse effect on the function of the donor foot in any patient (Table 2). Typical cases are illustrated in Figs. 6 and 7.

**Table 2:** Follow up outcomes

	Reconstructed						Donor		
Case	MHOQ (score)		DFE	2PD(mm)	CISS	Pain(VAS)	2PD(mm)	Pain(VAS)	C- MFPDI(score)
	Function	Appearance					Great / 2nd		
1	85	81.25	Excellent/Excellent	4/5	Mild	0	9/10	0	51
2	90	75	Excellent/Excellent	5/5	Mild	0	7/8	0	51
3	80	87.5	Excellent/Good	6/7	Mild	0	8/7	0	51
4	85	87.5	Good/Excellent	8/5	Mild	0	7/6	0	51
5	85	93.75	Excellent/Good	6/7	Mild	0	7/8	0	51
6	95	81.25	Excellent/Excellent	6/5	Mild	0	9/7	0	51
7	80	81.25	Excellent/Excellent	6/5	Mild	0	10/9	0	51
8	95	81.25	Excellent/Excellent	6/7	Mild	0	8/8	0	51
9	75	75	Good/General	7/6	Mild	0	7/8	0	51
10	80	87.5	Excellent/Excellent	4/6	Mild	0	8/6	0	51
11	90	81.5	Excellent/Excellent	7/5	Mild	0	10/8	0	51
12	80	93.75	Excellent/Excellent	5/7	Mild	0	9/7	0	51
13	75	81.25	Good/Good	7/9	Mild	0	8/9	0	51
14	95	75	Excellent/Excellent	6/5	Mild	0	7/8	0	51
15	90	87.5	Excellent/Excellent	4/5	Mild	0	8/9	0	51
16	95	93.75	Excellent/Excellent	4/5	Mild	0	9/10	0	51
17	90	87.5	Excellent/Excellent	5/6	Mild	0	8/10	0	51
18	85	87.5	Excellent/Good	5/4	Mild	0	10/8	0	51
19	80	81.25	Excellent/Good	6/6	Mild	0	7/9	0	51
20	90	93.75	Excellent/Excellent	7/5	Mild	0	10/7	0	51
21	90	81.25	Excellent/Excellent	6/8	Mild	0	8/8	0	51

MHOQ: Michigan Hand Outcomes Questionnaire; 2PD: Statically measured two-point discrimination; CISS: Cold Intolerance Severity Score; DFE: Dargan function evaluation; C-MFPDI: Chinese Manchester Foot Pain and Disability Index

## Discussion

### Structure of the fingers

Hand trauma is very common in the clinic. Due to the special structure of the fingers: the skin of the finger pulp has thick keratinized layer, and the deep surface has vertical fiber bundles connecting the skin with the superficial fascia, deep fascia, tendon sheaths and other deep structures, so that the skin of the finger pulp lack elasticity and is not easy to move, but is conducive to grasping, gripping, and holding objects, and the pulp of the finger has abundant nerve endings and sensory bodies; the dorsal side of the finger has thin subcutaneous soft tissues, which can easily lead to the skin defect when trauma occurs, exposing the tendons and bones, so it is very necessary to repair the middle and distal part of the finger when it is defective [1–3].

### Research status of flap repair for finger defects

Repair of finger injuries has always been a challenge for hand surgeons. In order to preserve the function, appearance, and sensation of the finger, a simple skin graft cannot meet the needs of finger injuries, and flap repair is often required. There are many methods of repair with varying results. Traditional repair methods include the V-Y flap, the adjacent finger flap, the abdominal flap, the island flap of the finger artery, and the dorsal fasciocu taneous flap, etc. The V-Y flap has a limited scope of repair and does not provide adequate coverage for defects larger than 1 cm2 in size. Neighboring finger flaps and abdominal flaps require prolonged forced positional immobilization, which is difficult for most patients to tolerate and does not allow reconstruction of sensation. The adjacent finger flap requires sacrifice of the integrity of the adjacent finger, but in this cases, the defects were located in the mid-distal segment of the adjacent finger, making this approach unfeasible. Abdominal flaps are also not optimal because of their bulky appearance, poor abrasion resistance, and severe hyperpigmentation. Finger artery island flaps have a reliable blood supply, but the sacrifice of one of the finger arteries makes the finger less cold-tolerant and does not allow for the reconstruction of sensation. In addition, the large size of the defects and the fact that some of the defects crossed the distal transverse finger stripe in the present patients made it impossible to perform the retrograde island flap technique in the finger arteries. The dorsal finger fasciocutaneous flap has poor blood supply, is prone to blistering, and has mild atrophy in the later stages of the flap, and in the case of large dorsal finger defects, this technique is not feasible. The dorsal metacarpal artery perforator flap has been reported by some authors to have a fair efficacy in multiple finger injuries, but it has a large postoperative scar in the donor area on the dorsal part of the hand, which is often a major obstacle in patients with high demands on the appearance of the hand. Patients with high demands on the appearance of the hand are often difficult to accept [7–13, 28].

### Flap from the foot is used to repair finger defects

The skin of the toes and fingers are similar tissues, and due to this natural similarity, tissue flap transplantation from the foot is often used to repair finger defects. By grafting the fibular side of the great toe or the tibial side of the second toe, it is possible to maximize the restoration of finger shape and sensation. This method keeps the donor area hidden, maintains constant vascularity, causes minimal damage to the donor foot, and does not affect walking. This approach aligns with the principle of tissue transplantation, aiming to restore and reconstruct the recipient area while minimizing traumatic loss to the donor area [14–16]. Therefore, some scholars have tried to solve this problem by designing a free first metatarsal dorsal artery bilobed flap with good results. Although the free first dorsal metatarsal artery bilobed flap had become a classic technique for repairing the proximal skin and soft tissue defects of two adjacent fingers, the combined transplantation of the fibular side of the great toe and the tibial side of the second toe with the first dorsal metatarsal artery as the pedicle for repairing the middle and distal defects of adjacent fingers has a lot of difficulties, because the transplanted fingers need to share the vascular pedicle, and there is the problem of insufficient vascular span, and it is often necessary to make two tissue flaps separately, which makes the surgical difficulty and the risk is higher. At the same time, when the toe tissue flap is cut, improper operation will often leave bone and tendon exposed, and it is not easy for the skin graft to survive, and it is often necessary to close the trauma by using a skin flap or amputation of the toe, and even if the skin graft survives, it is not wear-resistant, and the effect is less satisfactory.

In order to solve the problem of insufficient vascular span when the first and second toe tissue flaps share vascular pedicle for repairing mid-distal defects in adjacent fingers, many people have carried out a lot of explorations. Based on the type of anastomosis between the 1st dorsal metatarsal artery and the first plantar artery and the thickness of the arteries entering the great toe and the second toe, Xu YJ et al. used to cut off the plantar artery entering the lesser toe or the 2nd toe and then anastomosed with the plantar deep branch of the dorsal artery of the foot, respectively [16]. Although this method can increase the length of the vascular pedicle between one tissue flap, it is also necessary to do the vascular anastomosis in two places, and the surgical difficulty and risk is not reduced with the cutting of two tissue flaps respectively, which is not convenient for clinical promotion. Some scholars also use the free second toe of the first dorsal (basal) artery pedicle to reconstruct the fingers. at the same time carrying great toes fibular flap repair adjacent finger defects, the great fibular toe bottom artery surface with a skin bridge or suture into the skin tube, the same three weeks later cut off the skin bridge to break the tip [17]. Although the operation requires two times, it significantly reduces the risk and difficulty of the operation. However, cutting the skin between the toe webs of the 1st and 2nd toes or rolling it into a skin tube to cover the exposed vascular tip is easy to cause vascular compression or spasm due to swelling or exposed vascular tip, resulting in vascular complications of one side of the flap. At the same time, it is wasteful to the skin between the toe webs.

### Surgical exploration

In clinical practice, we have used the cell scaffold to cover the donor area after the foot flap has been cut in the clinical phase I. The skin is implanted after the dermis grows to cover the exposed tendon and phalanx about three weeks after surgery. This method greatly improves the survival rate and quality of the implanted skin and solves the problem of handling the donor area. At the same time, using of pedicle island flap skin grafting to repair wounds in the distal area in clinical practice, breaking the routine that the pedicle of the vascular pedicle flap needs to be buried under the skin. So that the repair has become more casual, and good results have been achieved. Combined with the previous experience, we use the free bilobed flap with the first dorsal (plantar) metatarsal artery pedicle repair the mid-distal defects in adjacent fingers, and apply the cell scaffold to cover the donor area of the foot, and at the same time, the excess cell scaffold is sewn into a leather tube to wrap the pedicle between the flaps. This surgery not only reduces the damage of the donor area and solves the embarrassment of the skin grafted of the donor area that is not abrasion-resistant and not easy to be viable, but also solves the defect that the bifurcation between the first and second toe base arteries is short, and the flap cannot be co-trunked to reach the middle distal segment through the subcutaneous tunnel. The use of a single vessel pedicle to supply blood to both traumas saves surgical time and reduces the risk and difficulty of the procedure.

### Mechanism of action of the cell scaffold

The silicone film on the outer layer of the cell scaffold mimics the epidermis, providing some resistance to infection and preventing water evaporation. Meanwhile, the inner layer consists of a collagen sponge with a porous reticular three-dimensional structure, capable of temporarily replacing dermal tissue. This structure can stimulate wound fibroblasts and capillary to grow and produce extracellular matrix, ultimately forming new tissue similar to the dermis. Overall, the cell scaffold’s structure closely resembles that of human skin, and the inner layer’s mesh scaffold creates a microenvironment conducive to the organized growth of capillaries, fibroblasts, and other components around the wound [29].

2–3 weeks later, the collagen will degrade and be replaced by new granulation tissue, forming visible pink granulation tissue in the clinic, which establishes blood circulation with the bone or tendon it covers, effectively covering the wound. When the cell scaffold provides enough matrix for skin grafting, we will perform a skin graft, which greatly improves the survival rate of the skin. The transplanted skin is elastic, durable, and scar-free.

### Advantages and disadvantages of surgery

#### Advantages

1. The trauma of adjacent fingers is repaired by a bilobed flap, only one set of blood vessels need to be anastomosed, which reduces the risk, time and difficulty of the operation. 2. The length of the exposed vessel pedicle between the bilobed flap can meet the the activity of finger splitting (two adjacent fingers can be separated in the coronal and sagittal plane), which is more conducive to the flexion and extension of the patient’s two injured fingers in a sagittal plane, so as to avoid the vascular pedicle being torn. 3. After the cell scaffold is covered, the granulation tissue under the wound surface of the donor area grows quickly, the quality is good, this improves the repair effect of the supply area. which avoids the trauma of toe amputation and flap repair in the donor area. 4. The vascular pedicle between the flaps is narrow, and it is easy to cut the pedicle and trim the shape, which can make the fingers get a satisfying look and good functionality. 5. The root of the toe nerve anastomosed with the nerve stumps of the fingers, giving a satisfactory restoration of sensation to the fingers.

#### Disadvantages

(1) The pedicle of the blood vessel between the flaps needs to be cut off twice. (2) The cell scaffold is expensive, requires a second surgical skin grafting. However, compared with other methods currently used in clinical practice, this method has obvious advantages and is worthy of clinical promotion. (3) Although the.

length of our vascular pedicle is longer than before, it still cannot meet the normal range of activity required for adjacent fingers. The movement of the two fingers needs.

to be synchronized to some extent, which may cause some patients to experience a temporary syndactylization effect. Therefore, we will promptly sever the pedicle to reduce the risk of syndactyly in patients. 4. The harvesting of the first dorsal ( plantar) metatarsal artery reduces the possibility of toe transfer on the same side due to the primary vascular deficiency during toe transplantation. (Table 3)

**Table 3:** Advantages and disadvantages

Advantages	Disadvantage
1. Using a bilobed flap for adjacent finger trauma reduces surgical complexity by requiring fewer blood vessel connections. 2. The length of the exposed vessel pedicle between the flaps allows for finger movement and helps prevent damage to the vascular pedicle. 3. Rapid granulation tissue growth in the donor area after covering the cell scaffold improves repair without the need for toe amputation or additional flap repair. 4. The narrow vascular pedicle between the flaps allows for easy trimming, resulting in aesthetically pleasing and functional fingers. 5. Nerve anastomosis provides satisfactory sensation restoration.	1. The blood vessel pedicle between the flaps needs to be cut twice. 2. The cell scaffold is costly and requires a second skin grafting surgery. 3. The vascular pedicle, although longer, cannot fully support normal finger movement, potentially causing temporary syndactylization. 4. Harvesting the first dorsal (plantar) metatarsal artery reduces the possibility of same-side toe transfer due to vascular deficiency during toe transplantation.

### Intraoperative and postoperative points

In order to obtain a better clinical effect, we should pay attention to the following points: (1) The donor area of the foot should completely stop bleeding, rinse the wound with normal saline, and try to maintain the sterility of the wound in the surgical area (2). For larger wounds, the cell scaffold needs to be compressed to ensure good contact between the cell scaffold and the wound (3). The time of the skin graft should be 3 weeks after the phase I surgery. When the silica gel film separates from the collagen sponge layer, the amount of granulation tissue is sufficient and turns from dark red to pink (4). Generally, 7–9 days postoperatively, after the risk period of the flap blood vessels, the patients should be actively instructed to carry out finger flexion and extension exercises to promote the maximum functional recovery of the joint. (Table 4)

**Table 4:** Intraoperative and postoperative points

POINTS
(1) Stop bleeding in the donor area, rinse the wound with saline, and maintain sterility in the surgical area.
(2) For larger wounds, compress the cell scaffold to ensure good contact with the wound.
(3) Skin graft should be done 3 weeks after phase I surgery, when granulation tissue is sufficient and turns from dark red to pink.
(4) After 7–9 days post-surgery, instruct patients to perform finger exercises for joint recovery.

There are certain limitations in this article: (1) It is a single-center study and has a small sample size. (2) Some cases have a short follow-up period. Further multicenter large-scale prospective studies are necessary to further confirm the efficacy and safety of this procedure. Despite some of the limitations, we believe that our study is still instructive.

## Conclusion

The surgical procedure of combined with the first dorsal (plantar) metatarsal artery pedicle free bilobed flap with a cell scaffold for the repair of mid-distal adjacent fingers defect is highly satisfactory. This approach helps the injured fingers to achieve good function, sensibility and appearance, while also achieving satisfactory results in the donor areas.

## Data Availability

No datasets were generated or analysed during the current study.

## Abbreviations

MHOQ: Michigan Hand Outcomes Questionnaire
DFE: Dargan Function Evaluationtwo-point discrimination
2PD: Two-point discrimination
CISS: Cold Intolerance Severity Score
VAS: Visual analogue score
D- MFPDI: E- Chinese Manchester Foot Pain and Disability Index
LMADF: Left middle and index fnger
RMADF: Right middle and index finger
RMARF: Right middle and ring finger
LMARF: Left middle and ring finger
LRALF: Left ring and little finger
M: Male
F: Female

## References

[1] Chen C, Tang P, Zhang L (2015). Reconstruction of a large soft-tissue defect in the single finger using the modified cross-finger flap. J Plast Reconstr Aesthet Surg.

[2] Matsui J, Piper S, Boyer MI (2014). Nonmicrosurgical options for soft tissue reconstruction of the hand. Curr Rev Musculoskelet Med.

[3] Neill BC, Roberts E, Tolkachjov SN (2021). Reconstructive options for cutaneous dorsal hand defects. Int J Dermatol.

[4] Lim JX, Chung KC, Advancement VY (2020). Thenar Flap, and cross-finger flaps. Hand Clin.

[5] Zhang J, Yao H, Mo J (2022). Finger-inspired rigid-soft hybrid tactile sensor with superior sensitivity at high frequency. Nat Commun.

[6] Gupta P, Lenchik L, Wuertzer SD, Pacholke DA (2015). High-resolution 3-T MRI of the fingers: review of anatomy and common tendon and ligament injuries. AJR Am J Roentgenol.

[7] Feng SM, Gu JX, Liu HJ (2013). Treatment of distal fingertip degloving injuries using a cross-finger flap based on the dorsal branch of the proper digital artery at the middle phalanx. J Reconstr Microsurg.

[8] Aydin HU, Savvidou C, Ozyurekoglu T. Comparison of Homodigital Dorsolateral Flap and Cross-Finger Flap for the Reconstruction of Pulp Defects. J Hand Surg Am. 2019;44:616.e1-616.e7. 10.1016/j.jhsa.2018.09.006 30366735

[9] Jia Z, Liang G, Duan C, Zhang Z, Guo Y, Teng Y (2022). Flow-through Dorsoulnar Perforator Flap repair of soft-tissue defect of the finger with Segmental Digital arterial defect. J Coll Physicians Surg Pak.

[10] Matei IR, Bumbasirevic M, Georgescu AV (2019). Finger defect coverage with digital artery perforator flaps. Injury.

[11] Tan RES, Kyi YW, Chong AK, Sebastin SJ. Pedicled Osteo-Onchyocutaneous Island Flap for Finger Macrodactyly: A Review of Literature. J Hand Surg Am. 2022;47:588.e1-588.e8. 10.1016/j.jhsa.2021.04.021 34078548

[12] Wu G, Zhang Z, Zhang F, Zhang Y, Wang Q, Yu W (2022). The free flap based on a single proximal perforator of the radial artery: ultrasonography study and clinical applications in reconstruction of soft tissue defects in finger. Eur J Med Res.

[13] Tang L, Zhou X, Zou Y (2022). Combined great toe dorsal nail-skin flap and medial plantar flap for one-stage reconstruction of degloved finger. Injury.

[14] Zhang X, Wang Z, Ma X (2022). Repair of finger pulp defects using a free second toe pulp flap anastomosed with the palmar vein. J Orthop Surg Res.

[15] Cheng LF, Lee JT, Wu MS (2019). Lateral toe pulp flap used in Reconstruction of Distal dorsal toe defect: Case Report and Review of the literature. Ann Plast Surg.

[16] Xu YJ, Shou KS, Rui YJ (2006). Reconstruction of thumb and index finger by combined transplantation of big toe wrap-around flap and the second toe. Chin J Hand Surg.

[17] Lin H, Li SH, Cong H, Wang XJ (2007). Application of the first dorsal metatarsal artery pedicle bicuspid tissue flap in finger reconstruction and repair. Chin J Microsurgery.

[18] Wang CY, Chiao HY, Chou CY (2017). Successful salvage and reconstruction of a finger threatened by Vibrio vulnificus necrotising fasciitis using fenestrated-type artificial dermis and three steps of topical negative pressure wound therapy. Int Wound J.

[19] Namgoong S, Jung JE, Han SK, Jeong SH, Dhong ES (2020). Potential of tissue-Engineered and Artificial Dermis grafts for Fingertip Reconstruction. Plast Reconstr Surg.

[20] Hou Q, Zeng LR, Wang LX (2011). The adjacent digital artery island flap repairs the soft tissue defect of the finger skin. Chin J Plast Surg.

[21] Hao Z (2020). Application of Pelnac® Artificial Dermis combined with VSD in the repair of Limb wounds. J Invest Surg.

[22] Liu T, Qiu C, Ben C, Li H, Zhu S (2019). One-step approach for full-thickness skin defect reconstruction in rats using minced split-thickness skin grafts with Pelnac overlay. Burns Trauma.

[23] Waljee JF, Kim HM, Burns PB, Chung KC (2011). Development of a brief, 12-item version of the Michigan Hand Questionnaire. Plast Reconstr Surg.

[24] Dargan EL (1969). Management of extensor tendon injuries of the hand. Surg Gynecol Obstet.

[25] Agarwal P, Mukati P, Kukrele R, Sharma D (2021). Simple indigenous two-point discrimination testing device. Neurol India.

[26] Magistroni E, Parodi G, Fop F (2020). Cold intolerance and neuropathic pain afterperipheral nerve injury in upper extremity. J Peripher Nerv Syst.

[27] Erh BXY, He HG, Carter KF (2019). Validation of the Chinese Manchester foot pain and disability index (C-MFPDI) among patients with inflammatory arthritis. J Foot Ankle Res.

[28] Chang DH, Hsieh CY, Chang CW, Chang KC, Chan CL (2021). Using dorsal metacarpal artery Perforator Flap in the soft tissue Reconstruction of traumatic finger defects: a single-center study. Ann Plast Surg.

[29] Wosgrau AC, Jeremias Tda S, Leonardi DF, Pereima MJ, Di Giunta G, Trentin AG (2015). Comparative experimental study of wound healing in mice: Pelnac versus Integra. PLoS ONE.

